# “You Win, You Buy”—How Continuous Win Effect Influence Consumers' Price Perception: An ERP Study

**DOI:** 10.3389/fnins.2018.00691

**Published:** 2018-10-05

**Authors:** Qingguo Ma, Linanzi Zhang, Manlin Wang

**Affiliations:** ^1^School of Management, Zhejiang University, Hangzhou, China; ^2^Institute of Neuromanagement Science, Zhejiang University of Technology, Hangzhou, China; ^3^Business School, Ningbo University, Ningbo, China; ^4^Academy of Neuroeconomics and Neuromanagement, Ningbo University, Ningbo, China; ^5^School of Management, Guizhou University, Guiyang, China

**Keywords:** continuous win effect, price perception, event-related potentials, P2, P300, LPP, neuromanagement, neuromarketing

## Abstract

Price played an important role in most purchases. Buying behavior was strongly determined by consumers' price expectations. Emotion as a research hotspot was demonstrated to be ubiquitous in marketing and influenced purchase processing as well. This study addressed interests upon whether emotion arousal would influence consumers' price perceptions and their willingness to purchase. Compared to such emotion researches which normally adopted emotional pictures as priming stimuli, we creatively employed a two-player “Finger Play” (FP) game without monetary gains or losses to arouse subjects' emotion in the experiment. A 2 (FP Game Results: Continuous Win vs. Continuous Lose) by 2 (Price Conditions: High Price vs. Low Price) Event-Related Potentials (ERPs) experiment was designed to investigate whether game results would arouse different emotions and influence subjects' perception of product price. Both behavioral and ERP results indicated that subjects' price perception was deeply impacted by emotions induced from continuous win/lose experiences.

## Introduction

Price played an important role in most purchases. Buying behavior was strongly determined by consumers' price expectations and the extent to which real prices violated those expectations (Schaefer et al., 2016). The ratio and trade-off between quality and price constituted a value-for-money conceptualization (Carvens et al., 1988; Monroe, 1990; Sweeney and Soutar, 2001). Normally, customers mostly avoided excessive prices products in addition to those deeply preferred ones (Knutson et al., 2007) and their expectations of higher quality at higher prices could be self-fulfilled if sellers did not charge higher prices for lower quality products (Gerstner, 1985). According to Gerstner (1985), consumer might understand the demand-related quality information or supply-related quality information of products through price (Gerstner, 1985). Having insight into of consumers' price perception and the influence factors during the purchase process became an important research emphasis of marketers and academicians.

Distinct components of the individuals' purchase decision processes might be difficultly separated and characterized by conventional research methods, while neuroscience and neuromanagement methods offered the hope (Knutson et al., 2007). Researches of consumer neuroscience draw heavily on the extant literatures in multiple areas since the very first published consumer neuroscience article which discussed how brand affected the experience of consumption (McClure et al., 2004; Smidts et al., 2014). Most works paid attention to products and price, as well as brands (Knutson et al., 2007; Plassmann et al., 2008, 2012; Smidts et al., 2014). For example, Knutson et al. (2007) investigated neural predictors of purchase processing and indicated that ‘excessive prices activated the insula and deactivated the mesial prefrontal cortex (MPFC) prior to the purchase decision’ using fMRI technology. Karmarkar et al. (2015) examined the sequence-dependent effects of price and product information on decision-making process using fMRI methods and suggested that price primacy highlighted considerations of product worth, and could thereby influence purchasing in conclusion (Sacré et al., 2016).

Some academicians draw their research interests on the relevance of emotion and marketing in the studies of perspective of cognitive science and consumer neuroscience (Mick and Fournier, 1998; Shiv and Fedorikhin, 1999; Chaudhuri and Holbrook, 2001; Belk et al., 2003; Henning-Thurau et al., 2006; Yan et al., 2018). Emotion was ubiquitous in marketing and influenced purchase processing, measured effects of marketing-mix tactics, as well as consumer welfare (Baogozzi et al., 1999). Neuroscientists also illustrated that decision making involved not only the cold-hearted calculation of expected utility but also more subtle and sometimes depending critically upon emotion (Shiv and Fedorikhin, 1999). The significant advances in the study of emotion and decision making had been seen obviously (Shiv, 2007). Advances had occurred in the study of task-induced affect, integral affect, and anticipatory affect as well (Luce, 1998; Shiv and Nowlis, 2004; Nowlis and Shiv, 2005).

Studies on emotion were mainly carried out in the following three aspects: emotion valence (unpleasant-to-pleasant), emotional arousal (low-to-high), and paradigms to evoke emotional process. Most affective ERP researches adopted stimuli from the International Affective Picture System (IAPS) which contains pictures rated with valence category (pleasant and unpleasant) and arousal level (low to high) on a nine-point scale (Lang et al., 1999; Olofsson et al., 2008). Neural imaging studies further demonstrated the separate attributes of emotion as positive and negative (Grodd et al., 1995; Schneider et al., 1997). Lifshitz (1966) found that pleasant and unpleasant pictures induced a positive-going waveform at about 350–450 ms after stimulus onset compared to neutral ones back in the sixty's of twentieth century (Lifshitz, 1966). Schupp et al. (2000) also indicated larger late positive potentials (LPP) elicited when subjects saw pleasant and unpleasant pictures rather than neutral ones. Additionally, the latter portion of ERP waveform LPP indicated elevated positivity to high-arousing stimulation (Cuthbert et al., 2000).

Normally, most people love to win and hate to lose when they play a game. Some findings of psychological and mental researches explained the phenomenon by considering winning as a reward while losing as a punishment for people (Robbins and Everitt, 2007; Salamone et al., 2007; Tomer et al., 2014). Humans constantly chose actions based on the balance between the desire for pleasure and aversion to punishment (Tomer et al., 2014). Many of the researches on winning and losing concentrated upon competitions or gambling games and pointed out that winning or losing would influence a wide range of social and personal behaviors (Elliot and Covington, 2001; Smith et al., 2009; Zysberg and Kimhi, 2013; Doron and Gaudreau, 2014; Dugatkin and Reeve, 2014; Smidts et al., 2014; Xu and Harvey, 2014; Sacré et al., 2016). The neural activity modulated significantly between win and lose trials in the anterior insula and gamma-band activity increased around 500 ms after the show computer cards for win trials (Sacré et al., 2016). Yeung et al.'s research Yeung and Sanfey (2004) examined the properties of P300 and feedback negativity components which were unpredictably associated with monetary gains and losses of variable magnitude in a simple gambling game. According to their findings, P300 was sensitivity to the reward value of alternative, non-selected stimuli. The two-factor theory of emotion explained the process of human emotional awakening (Schachter and Singer, 1962; Burns and Corpus, 2004) and suggested that a deviant situation such as a winning or losing streak in sports games had the potential to regulate the human's emotional state or choice behavior (Ayton and Fischer, 2004; Attali, 2013).

This study addressed interests on the role of emotion in consumers' price perception. Since winning or losing might make people have positive or negative feelings (pleasant vs. unpleasant), whether a competitive game could be adopted as the emotional priming paradigm instead of affective pictures to do the investigation. With our best knowledge and in order to strengthen subjects' sense of presence and authenticity, we creatively applied a two-player simple game—“Finger Play” (FP) known by nearly everyone involved no monetary gains or losses as the emotion priming stimuli, resulting a 2 (FP Game Results: Continuous Win vs. Continuous Lose) by 2 (Price Levels: High Price vs. Low Price) event-related potentials (ERPs) experiment designed to explore two research issues: (1) whether game results without monetary gain/loss would arouse subjects' different emotions; (2) If it would be, how the emotions impact subjects' perception of product price and their buying behavior. After the ERP experiment, participants were asked to rank a preference score of products chosen for this study to confirm that conditions of products attributes were controlled.

## Materials and methods

### Participants

Twenty-six healthy, right-handed students (nine female) recruited at Zhejiang University participated in this study, age from 19 to 27 years (*Mean* = 22.07 years, *SD* = 1.9 years). All subjects were native Chinese speakers, had normal or corrected-to-normal vision, and did not have any history of neurological disorders or mental diseases. This study was approved by the Neuromanagement Laboratory Ethics Committee at Zhejiang University. Informed consents were obtained from subjects prior to the formal experiments. Data from three subjects were discarded for excessive recording artifacts, resulting 23 (eight female) valid subjects (aged 19–27 years, *Mean* = 22.04 years, *SD* = 1.92 years) were included in the final data analysis.

### Stimulus material

Sixty hard-disc pictures which had same color and similar shape were used in this study. All the pictures were obtained from the Internet and the brands were erased. In ERP experiment, two groups of random number were generated from Excel of Windows Office 2007 and each hard-disc picture was paired with a high price set (from 500 to 700 RMB) and a low price set (from 200 to 400 RMB). Product pictures were equally and randomly divided into four groups, group 1 had 15 pictures corresponded with continuous win situation, group 2 also had 15 pictures corresponded with continuous lose situation, group 3 and group 4 had 30 pictures linked to other situations (e.g., two draws/one win-one draw/one lose-one draw etc.). Each product picture was paired with two prices (high price vs. low price), resulting doubled the number of total pictures. Every product picture with price presented twice and in a random sequence during the whole experiment. In preference rating experiment, same pictures used in ERP experiment were obtained, however, price information was erased in each picture. All the pictures were adjusted to a uniform size (10 by 11 cm, 300 by 332 pixels) and gray-processed by Photoshop software to ensure consistency in the background, brightness, contrast and color.

### Experiment procedure

Experiment instruction was shown to participants on paper handouts at the very beginning. Subjects were seated comfortably in a dimly lit, sound-attenuated and electrically shielded room. Experimental stimuli were presented centrally on a computer screen at a distance of 100 cm from each subjects' face. A keypad was provided to the participants to make their choices. Each subject had three practice trials to become familiar with the experimental procedure before the formal experiment.

**Figure 1:**
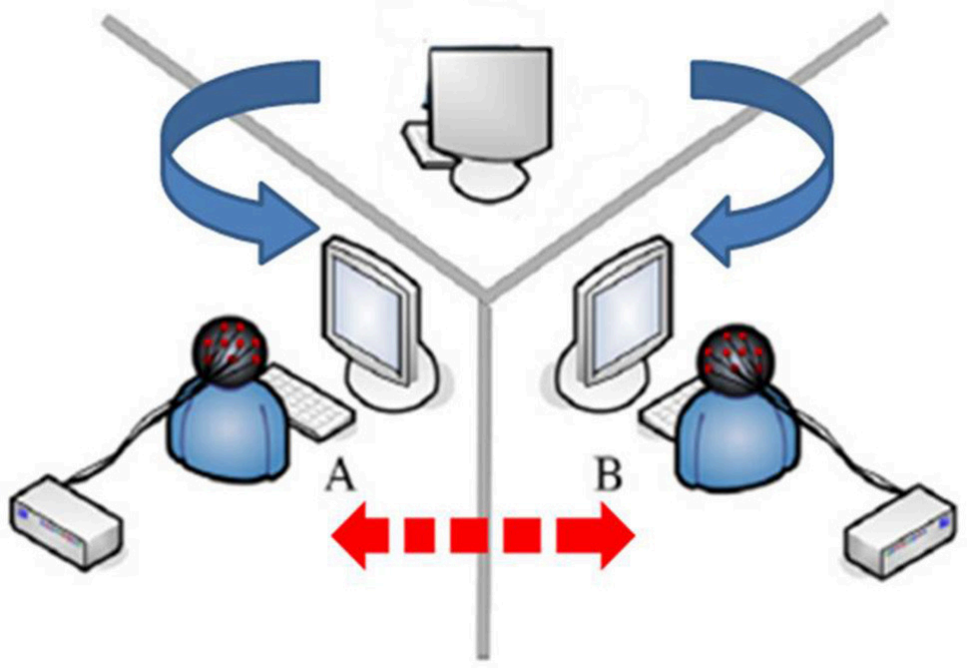
Experimental schematic diagram. Two participants joined in the ERP experiment. They met firstly and were informed to play FP game with each other before entering in the different rooms. Two experimenters started the experimental program together with a 3 beats countdown. Participants actually play the game with the computer programs.

In order to construct subjects' sense of presence and authenticity, we referred to the experiment schematic diagram of Ma et al. (2011) with some changes on the basis of our study purposes (see Figure 1). In their research, three persons (two friends and one stranger) were recruited at once to do a gamble task together and evaluated their empathic responses to others' gains or losses (Ma et al., 2011); in our research, two subjects (strangers) were recruited at once and they did the ERP experiment in different rooms at the same time. All subjects were informed that they planned to buy a hard-disc on a shopping website. The shape, quality, function and color of these hard-discs were no significant differences; the only different information was the price. They were required to decide whether to add a hard-disc to cart in every trial, however, they did not need to actually buy it in the end. Moreover, we simulated the FP game as an online interactive activity developed by the shopping website before they browsed the products. All participants were informed that if they played the FP game, they would receive a coupon for shopping next time. Further, in our experiment, every two-participant was told that they were the randomly paired customers to play the game together before they searched the hard-disc. To be worth mentioning, the game results were actually operated by the computer program (wrote by E-Prime 2.0) to confirm that there would be sufficient effective trials for each condition. From an ethical perspective, all participants were informed that they actually played the game with computer program in the end of the experiment.

**Figure 2:**
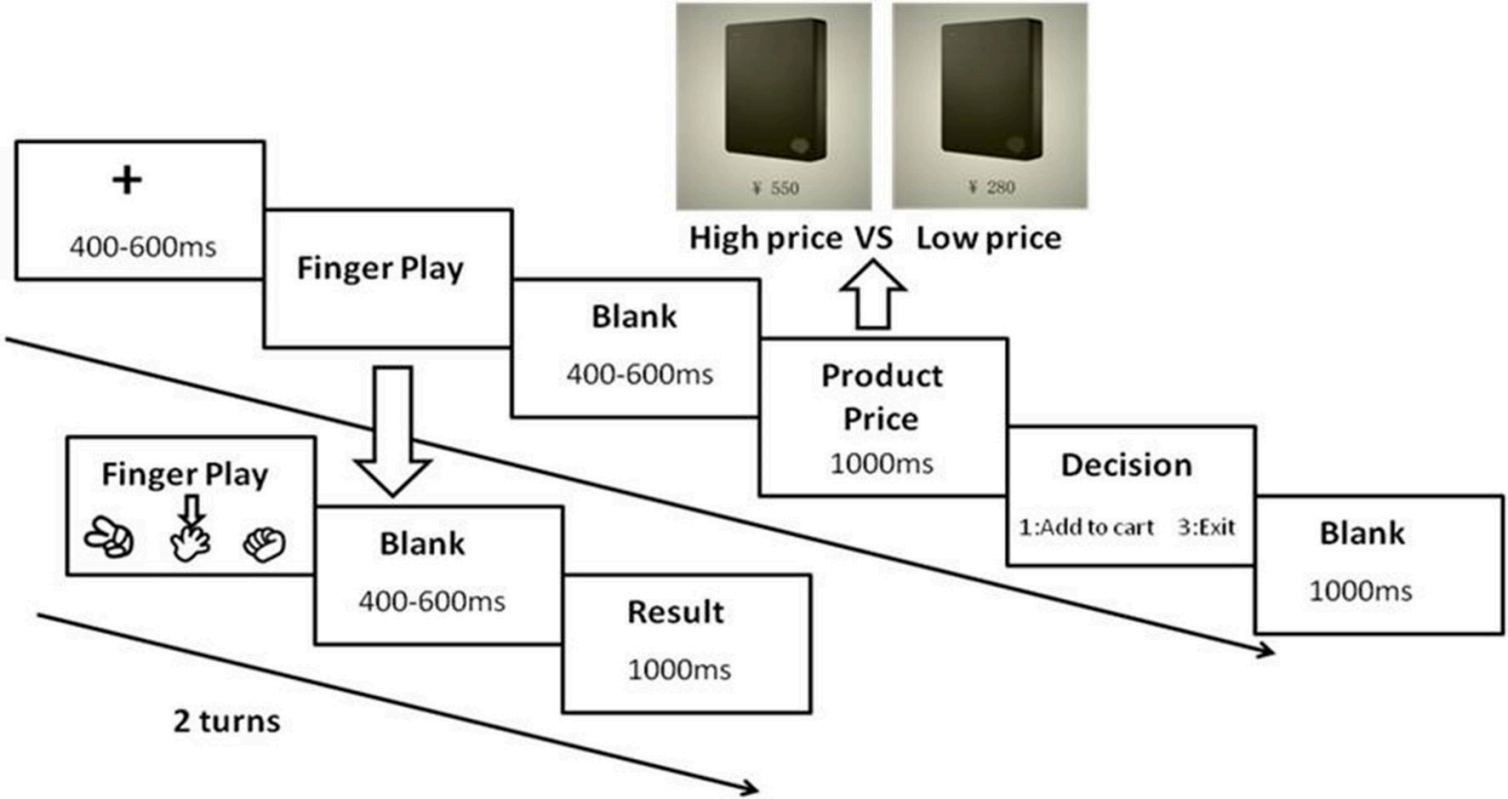
A single trial of the ERP experiment.

Figure 2 indicates a single trial of ERP experiment. First, a randomly 400–600 ms fixation appeared on a gray screen. Then, the FP frame presented until the end of subjects' choice (key 1 and 3 to move the arrow, key 2 to confirm the choice; the rules of FP: scissors > paper > stone > scissors). The game result showed for 1,000 ms after a randomly blank (400–600 ms). After two turns of the game, a hard-disc picture with high or low price continuously presented after another randomly 400–600 ms blank and lasted 1,000 ms. Participants subsequently made their decision whether adding this hard-disc to cart or not with a provided keypad (key 1 = add to cart, key 3 = not add to cart; half subjects pressed key 3 to add and key 1 to exit). A 1,000 ms blank showed at the end of each trial.

There were two blocks in this experiment, every subject had 180 trials (90 trials for each block) in total [include 60 trials of continuous win situation (30 for high price products and 30 for low price products), 60 trials of continuous lose situation and 60 trials of other situations]. Participants took a rest about 3–5 min during the two blocks.

**Figure 3:**
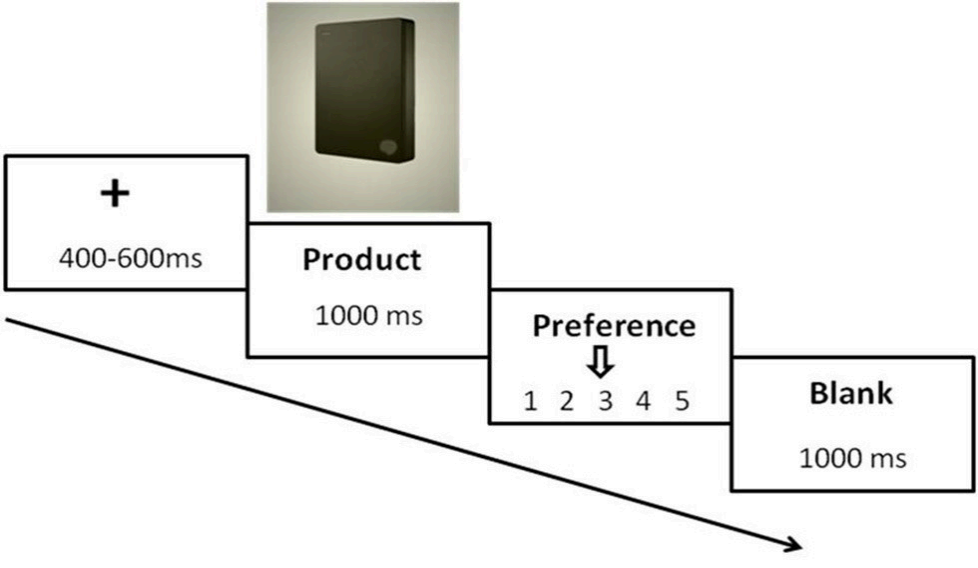
A single trial of preference rating experiment.

When subjects finished the ERP experiment, they would have a 10 min rest. After the rest, all participants were asked to do the preference rating experiment (see Figure 3).

Figure 3 showed the procedure of the preference rating experiment. FP was removed and hard-disc pictures without price information were presented directly. A randomly 400–600 ms fixation came first, then the hard-disc picture was onset for 1,000 ms, participants used a provided keypad to rank their preference degree of the product (key 1 and 3 to move the arrow, key 2 to confirm the preference score). Likert scale with 5 points (from 1 = “not like it at all” to 5 = “extremely like it”) was used as rating method. Each hard-disc pictures randomly appeared twice in this experiment, resulting 120 trials in total.

### Electrophysiological recordings

Scalp voltages were recorded (band-pass 0.05–70 Hz, sampling rate 500 Hz) from a 64-channel electro-cap according to the 10/20 system and using a NeuroScan SynAmps 2 Amplifier (Scan 4.5, Neurosoft Labs, Inc. Virginia, USA). A cephalic (forehead) location was connected as the ground. EEGs were off-line re-referenced to the average of the left and the right mastoids. Horizontal Electrooculogram (EOG) was recorded at the left vs. right orbital rim while vertical EOG was recorded supra and infra-obitally at the left eye. The electrode impedance was maintained below 5 kΩ during the recording.

### Data analysis

For the behavioral data, descriptive statistics and *T*-test were used to analyze the ratio of adding to cart in the experiment. Further, a paired-samples *T*-test was adopted to analyze the preference degree ranking sores of hard-discs.

EEG data were pre-processed through the software NeuroScan 4.5 before the statistic analysis. EOG artifacts were corrected firstly, followed by digital filtering through a zero phase shift (low pass at 30 Hz, 24 dB/octave). The EEGs were segmented for 1,000 ms in each epoch, beginning 200 ms before and continuing until 800 ms after the onset of both the game results and products presentations. The entire epoch was then baseline-corrected by the 200 ms interval prior to the stimulus onset. Trials that contained amplifier clipping, bursts of electromyography activity, or peak-to-peak deflection exceeding ±80 μV were excluded from the final average. Matlab R2015b was obtained to generate topographic maps for each condition.

The statistic method repeated measures ANOVA was adopted to do the statistics analysis of ERP results. In our study, the dependent variables of ANOVA with the FP game results onset were the amplitudes of P300 and LPP, respectively, and the independent variables were the two levels of finger play results [Single Win vs. Single Lose (SW vs. SL)/Continuous Win vs. Continuous Lose (CW vs. CL)/Single Win vs. Continuous Win (SW vs. CW)/Single Lose vs. Continuous Lose (SL vs. CL)] and the electrodes with nine levels (C1, Cz, C2, CP1, CPz, CP2, P1, Pz, P2). The dependent variables of ANOVA with hard-disc onset were the amplitudes of P2 and LPP, respectively, and the independent variables were hard-disc with price in four groups [high price hard-discs paired to continuous win (CWHP) vs. low price hard-discs paired to continuous win (CWLP) vs. high price hard-discs paired to continuous lose (CLHP) vs. low price hard-discs paired to continuous lose (CLLP)] and the electrodes with six levels (F3, Fz, F4, FC3, FCz, FC4) for P2 and nine levels (C1, Cz, C2, CP1, CPz, CP2, P1, Pz, P2) for LPP.

Based on the visual observation, the mean amplitudes from 290 to 400 ms of the central-parietal P300 and the mean amplitudes of LPP (from 500 to 800 ms) were mainly analyzed to examine the neural process when subjects knew the finger play results. Nine electrode sites from central parietal (C1, Cz, C2, CP1, CPz, CP2, P1, Pz, P2) were chosen for the analysis of P300 and LPP components. ANOVA factors were stimulus type (two levels: SW vs. SL or CW vs. CL or SW vs. CW or SL vs. CL) and electrodes (nine levels: C1, Cz, C2, CP1, CPz, CP2, P1, Pz, P2).

When a hard-disc picture was onset, the peak amplitude of early P2 component (in the range of 150–220 ms) was analyzed through six electrode sites (F3, Fz, F4, FC3, FCz, FC4) and the mean amplitude of late LPP component (from 500 to 800 ms) was analyzed through nine electrodes sites (C1, Cz, C2, CP1, CPz, CP2, P1, Pz, P2). ANOVA factors were price levels corresponding with FP results (four levels: CWHP vs. CWLP vs. CLHP vs. CLLP) and electrodes [six levels (F3, Fz, F4, FC3, FCz, FC4) for P2 and nine levels (C1, Cz, C2, CP1, CPz, CP2, P1, Pz, P2) for LPP]. The Greenhouse-Geisser correction was applied in all statistical analyses when necessary.

## Results

### Behavioral results

According to our research purposes, we only analyzed the adding to cart ratio of hard-discs corresponded to continuous win or continuous lose results and the preference ranking scores of pictures linked to these two conditions.

#### Results for “adding to cart” task

**Figure 4:**
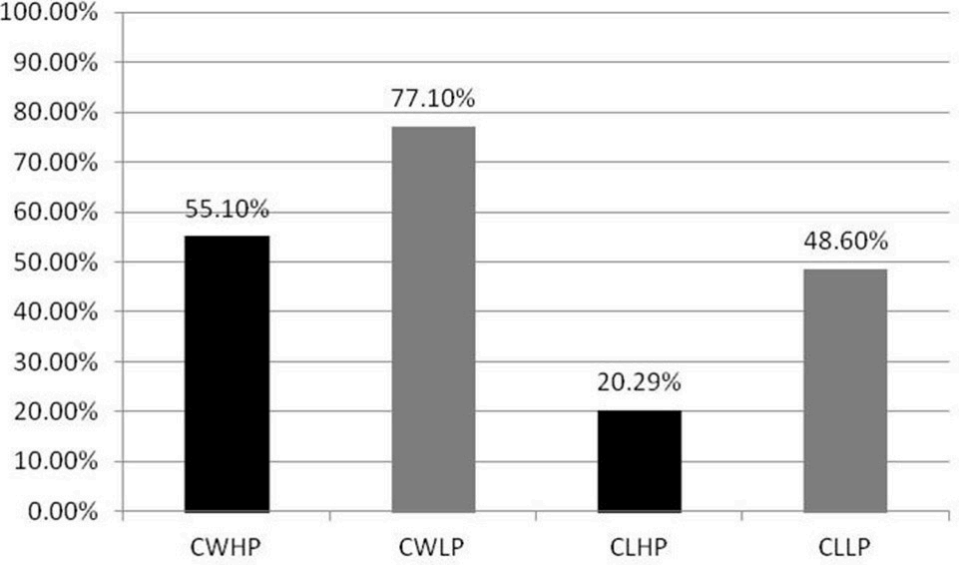
The adding to cart proportion in four situations [CWHP (Continuous Win-High Price), CWLP (Continuous Win-Low Price), CLHP (Continuous Lose-High Price), and CLLP (Continuous Lose-Low Price)].

Descriptive statistics was adopted to analyze the behavioral data. Figure 4 showed the choice results subjects made in the experiment. The adding to cart proportion of CWHP was 55.1% (*SD* = 0.36), while the proportion of CLHP was 20.29% (*SD* = 0.24). Compared with CLLP (*M* = 48.6%, *SD* = 0.31), the adding to cart proportion of CWLP was much higher (*M* = 77.1%, *SD* = 0.29). The statistical results of paired-samples *T*-test also indicated that there were significant differences of adding to cart proportion among these four products groups (*t*_*CWHP*−*CWLP*_ = 2.119, *p* = 0.046; *t*_*CLHP*−*CLLP*_ = 3.768, *p* = 0.001; *t*_*CWHP*−*CLHP*_ = 4.391, *p* < 0.01; *t*_*CWLP*−*CLLP*_ = 3.218, *p* = 0.004).

#### Results for preference rating task

A paired-samples *T*-test was obtained to analyze the preference ranking scores of the hard-discs. Without the FP priming stimuli, subjects saw the products without price information directly and ranked nearly no differences in scores of preferences between products linked to CW results and CL results in the ERP experiment (*Mean*_*Harddiscs*−*CW*_ = 2.96, *SD* = 0.369; *Mean*_*Harddiscs*−*CL*_ = 3.02, *SD* = 0.357), and the T test result was not significant (*t* = 0.835, *p* = 0.413).

### ERPS results

#### FP results onset

**Figure 5:**
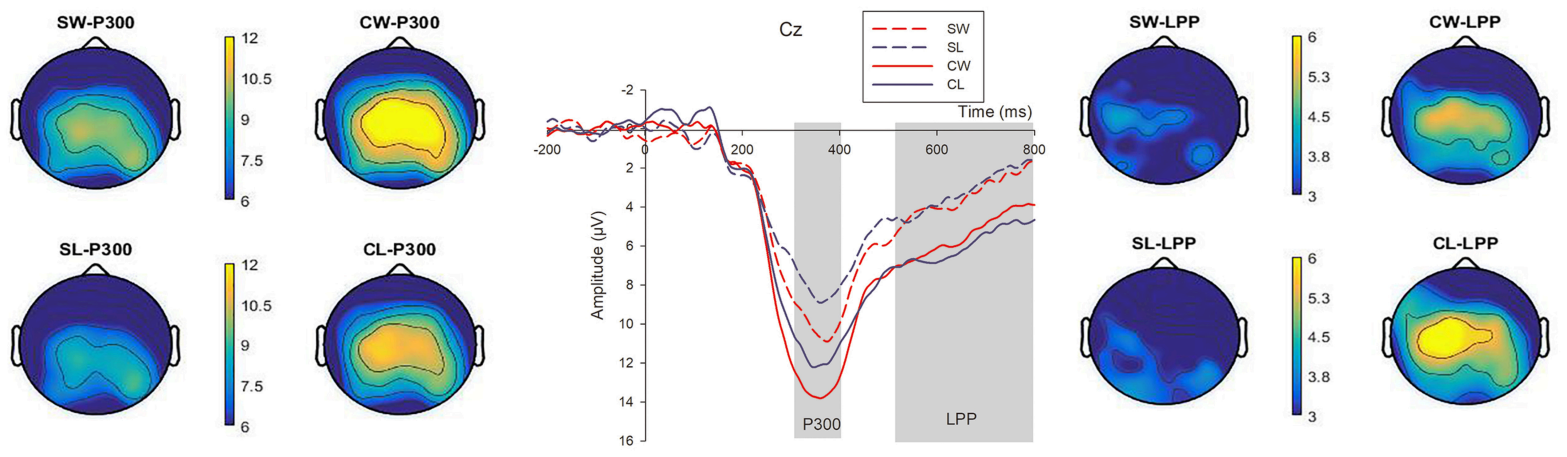
The ERP grand-average waveforms at Cz and topographical maps of P300 and LPP in four game results conditions [“SW” (Single Win), “SL” (Single Lose), “CW” (Continuous Win), and “CL” (Continuous Lose)]. The time window for P300 was 290–400 ms, while 500–800 ms for LPP. The bar of the topographical map ranges from 6–12 μV for P300 to 3–6 μV for LPP.

We compared three situations' scalp waves which included SW vs. SL, CW vs. CL and SW/SL vs. CW/CL when FP result was onset. P300 and LPP components were observed in the situation (see Figure 5 and Table 1).

**Table 1:** ANOVA Results of FP results onset.

**Source**		**Value**	***F***	**Hypothesis df**	**Error df**	**Sig**.
**P300 (290–400 ms)**						
SW vs.SL	Pillai's Trace	0.314	10.051^a^	1.000	22.000	0.004
	Wilks' Lambda	0.686	10.051^a^	1.000	22.000	0.004
	Hotelling's Trace	0.457	10.051^a^	1.000	22.000	0.004
	Roy's Largest Root	0.457	10.051^a^	1.000	22.000	0.004
CW vs. CL	Pillai's Trace	0.261	7.767^a^	1.000	22.000	0.011
	Wilks' Lambda	0.739	7.767^a^	1.000	22.000	0.011
	Hotelling's Trace	0.353	7.767^a^	1.000	22.000	0.011
	Roy's Largest Root	0.353	7.767^a^	1.000	22.000	0.011
CW vs. SW	Pillai's Trace	0.307	9.753^a^	1.000	22.000	0.005
	Wilks' Lambda	0.693	9.753^a^	1.000	22.000	0.005
	Hotelling's Trace	0.443	9.753^a^	1.000	22.000	0.005
	Roy's Largest Root	0.443	9.753^a^	1.000	22.000	0.005
CL vs. SL	Pillai's Trace	0.304	9.619^a^	1.000	22.000	0.005
	Wilks' Lambda	0.696	9.619^a^	1.000	22.000	0.005
	Hotelling's Trace	0.437	9.619^a^	1.000	22.000	0.005
	Roy's Largest Root	0.437	9.619^a^	1.000	22.000	0.005
**LPP (500–800 ms)**						
CW vs. SW	Pillai's Trace	0.188	5.091^a^	1.000	22.000	0.034
	Wilks' Lambda	0.812	5.091^a^	1.000	22.000	0.034
	Hotelling's Trace	0.231	5.091^a^	1.000	22.000	0.034
	Roy's Largest Root	0.231	5.091^a^	1.000	22.000	0.034
CL vs. SL	Pillai's Trace	0.230	6.584^a^	1.000	22.000	0.018
	Wilks' Lambda	0.770	6.584^a^	1.000	22.000	0.018
	Hotelling's Trace	0.299	6.584^a^	1.000	22.000	0.018
	Roy's Largest Root	0.299	6.584^a^	1.000	22.000	0.018

a *Means the exact statistic*.

##### P300

According to the statistical analysis, the main effect of FP results was significant (*F*_*SW*−*SL*−*P*300_ = 10.051, *p* = 0.004; *F*_*CW*−*CL*−*P*300_ = 7.767, *p* = 0.011; *F*_*CW*−*SW*−*P*300_ = 9.753, *p* = 0.005; *F*_*CL*−*SL*−*P*300_ = 9.619, *p* = 0.005). The amplitudes of SW and CW results were larger than SL and CL results. We also found larger P300 amplitudes of CW compared with SW, same as CL vs. SL (*Mean*_*SW*−*P*300_ = 9.57 μ*V, SD* = 1.6; *Mean*_*SL*−*P*300_ = 8.24 μ*V, SD* = 1.44; *Mean*_*CW*−*P*300_ = 11.996 μ*V, SD* = 1.66; *Mean*_*CL*−*P*300_ = 10.45 μ*V, SD* = 1.55).

##### LPP

The main effect of FP results was significant when we compared the CW vs. SW conditions as well as CL vs. SL conditions (*F*_*CW*−*SW*−*LPP*_ = 5.091, *p* = 0.034; *F*_*CL*−*SL*−*LPP*_ = 6.584, *p* = 0.018). Larger LPP amplitudes were elicited in CW and CL results compared with SW and SL results (*Mean*_*SW*−*LPP*_ = 3.32 μ*V, SD* = 0.91; *Mean*_*SL*−*LPP*_ = 3.44 μ*V, SD* = 1.12; *Mean*_*CW*−*LPP*_ = 4.84 μ*V, SD* = 0.88; *Mean*_*CL*−*LPP*_ = 5.42 μ*V, SD* = 1.04). However, the main effects of neither SW vs. SL nor CW vs. CL conditions were significant (*F*_*SW*−*SL*−*LPP*_ = 0.05, *p* = 0.823; *F*_*CW*−*CL*−*LPP*_ = 1.42, *p* = 0.246).

#### Hard-discs with high or low prices onset

According to the visual observation, the late positive potentials component (LPP) was mainly analyzed of the neural process. In addition, the early P2 was also observed when we compared the CWHP and CLHP conditions (see Table 2).

**Table 2:** ANOVA results of hard-discs with high or low price onset.

**Source**		**Value**	***F***	**Hypothesis df**	**Error df**	**Sig**.
**P2 (150–220 ms)**						
CWHP–CLHP	Pillai's Trace	0.167	4.398^a^	1.000	22.000	0.048
	Wilks' Lambda	0.833	4.398^a^	1.000	22.000	0.048
	Hotelling's Trace	0.200	4.398^a^	1.000	22.000	0.048
	Roy's Largest Root	0.200	4.398^a^	1.000	22.000	0.048
**LPP (500–800 ms)**						
CWHP-CLHP	Pillai's Trace	0.222	6.274^a^	1.000	22.000	0.020
	Wilks' Lambda	0.778	6.274^a^	1.000	22.000	0.020
	Hotelling's Trace	0.285	6.274^a^	1.000	22.000	0.020
	Roy's Largest Root	0.285	6.274^a^	1.000	22.000	0.020
**LPP Latency**						
CWLP–CLLP	Pillai's Trace	0.265	7.925^a^	1.000	22.000	0.010
	Wilks' Lambda	0.735	7.925^a^	1.000	22.000	0.010
	Hotelling's Trace	0.360	7.925^a^	1.000	22.000	0.010
	Roy's Largest Root	0.360	7.925^a^	1.000	22.000	0.010

a *Means the exact statistic*.

##### P2

**Figure 6:**
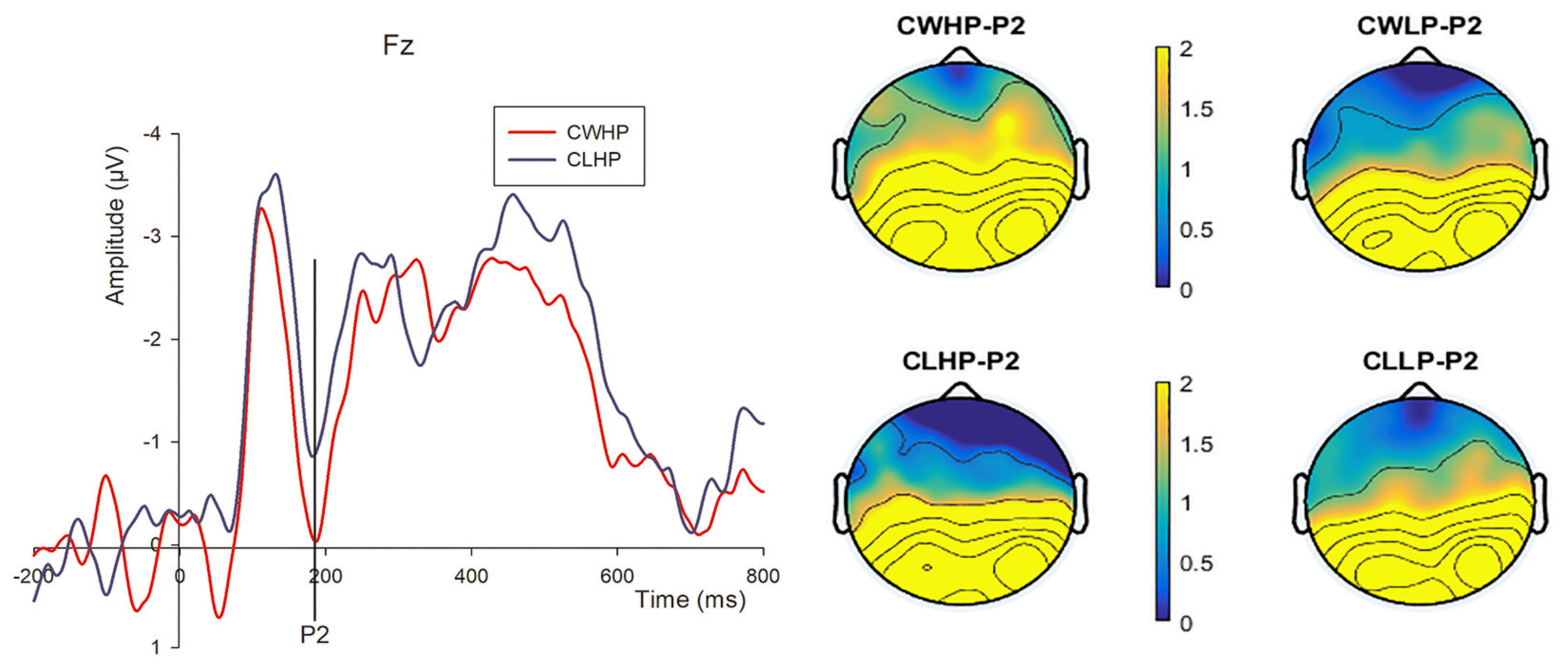
The comparison of the peak-average waveforms from 150 to 220 ms at Fz and topographical maps of P2 in CWHP and CLHP conditions. The bar of the topographical map ranges from 0 to 2 μV.

The main effect of Continuous Win compared with Continuous Lose in High Price condition was significant (*F*_*CWHP*−*CLHP*−*P*2_ = 4.398, *p* = 0.048) and larger peak amplitude was elicited in CWHP condition (*Mean*_*CWHP*−*P*2_ = 1.508 μ*V, SD* = 0.665; *Mean*_*CLHP*−*P*2_ = 0.561 μ*V, SD* = 0.678). The latencies of these two conditions had no significant difference (*F*_*CWHP*−*CLHP*−*P*2*Latency*_ = 0.552, *p* = 0.465; *Mean*_*CWHP*−*P*2*Latency*_ = 186.87 ms, *SD* = 3.852; *Mean*_*CLHP*−*P*2*Latency*_ = 189.58ms, *SD* = 4.037) (see Figure 6). However, we did not find significant differences across other conditions (*F*_*CWHP*−*CWLP*−*P*2_ = 1.186, *p* = 0.288; *F*_*CLHP*−*CLLP*−*P*2_ = 0.947, *p* = 0.341; *F*_*CWLP*−*CLLP*−*P*2_ = 0.042, *p* = 0.84; *Mean*_*CWLP*−*P*2_ = 0.978 μ*V, SD* = 0.645; *Mean*_*CLLP*−*P*2_ = 1.064 μ*V, SD* = 0.731).

##### LPP

**Figure 7:**
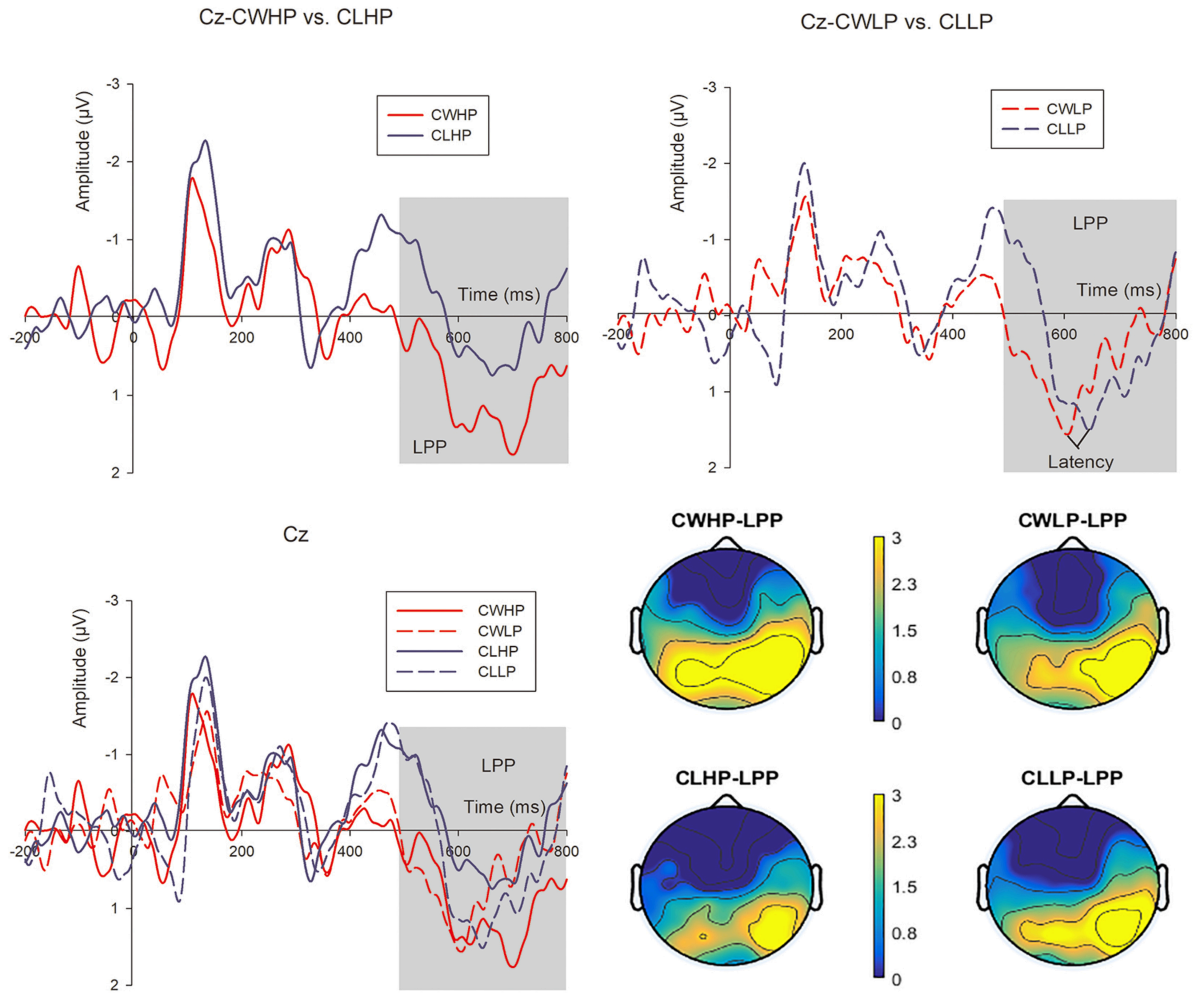
The LPP grand-average waveforms from 500 to 800 ms at Cz and the topographical maps in the four conditions: CWHP, CWLP, CLHP, and CLLP. The bar of the topographical map ranges from 0 to 3 μV.

The main effects of CWHP vs. CLHP was significant (*F*_*CWHP*−*CLHP*−*LPP*_ = 6.274, *p* = 0.02). Larger mean amplitudes from 500 to 800 ms of late positive potentials were observed of CWHP compared with CLHP (*Mean*_*CWHP*−*LPP*_ = 2.364 μ*V, SD* = 0.972; *Mean*_*CLHP*−*LPP*_ = 1.138 μ*V, SD* = 0.879). We did not find significant differences of LPP effects among CWLP vs. CLLP, CLHP vs. CLLP and CWHP vs. CWLP conditions (*F*_*CWLP*−*CLLP*−*LPP*_ = 0.16, *p* = 0.693; *F*_*CLHP*−*CLLP*−*LPP*_ = 0.136, *p* = 0.716; *F*_*CWHP*−*CWLP*−*LPP*_ = 0.022, *p* = 0.884; *Mean*_*CWLP*−*LPP*_ = 1.764 μ*V, SD* = 0.812; *Mean*_*CLLP*−*LPP*_ = 1.558 μ*V, SD* = 0.784). However, we examined the latency of peak amplitudes of LPP during the 500–800 ms time duration and found significant differences between CWLP and CLLP conditions (*F*_*CWLP*−*CLLP*−*LPPLatency*_ = 7.925, *p* = 0.01; *Mean*_*CWLP*−*LPPLatency*_ = 609.45 ms, *SD* = 13.482; *Mean*_*CLLP*−*LPPLatency*_ = 662.37 ms, *SD* = 12.636; see Figure 7).

## Discussion

There were two main purposes of this study. One was to examine whether the two-player “Finger Play” game without monetary gains or losses could be an emotional priming paradigm using in an event-related potentials experiment. The other was to investigate the neural process of emotions influencing subjects' price perceptions and their buying behavior. According to our experiment results, these two objectives were both achieved through the behavioral data and ERPs measures.

A growing consensus in recent years witnessed that there were tightly intertwined of affective and cognitive processes (Olofsson et al., 2008; Winkielman and Gogolushko, 2018). Both psychological function and neural substrates offered evidences to understand the mechanisms of this connection (LeDoux, 2012; Pessoa, 2013, 2015). Subsequent studies discovered the influence of visually suppressed affective pictures including face expressions or emotional words on a variety of reactions, behaviors, judgments and decisions. Emotion-related researches normally adopted affective pictures or emotional words as the priming stimuli (Begleiter et al., 1967; Sweeny et al., 2009; Axelrod et al., 2015; Diéguez-Risco et al., 2015; Zhang et al., 2015; Chanes et al., 2018). Existing research findings expounded that winning or losing might induce positive or negative emotions in sports games or gambling (Lole et al., 2013; Doron and Gaudreau, 2014; Tomer et al., 2014; Sacré et al., 2016; Kamei et al., 2018), yet few studies adopted it as an emotional priming and integrated it to investigate with other topics especially business or marketing issues. To our best knowledge, we firstly employed a two-player FP game as an emotional priming paradigm and integrated it into a price perception investigation. The reason we chose FP game was because it was easily understood and nearly everyone knew the playing rules. In addition, monetary gains or losses were out of consideration as it might cause other complexity mental issues which were not our research emphasis. More important, we recruited two subjects to do the ERP experiment in different rooms and recorded the data at the same time. Those two participants signed on the informed consents and read the experimental instructions together before the formal experiment. The reason for doing that was to create and strengthen the sense of authenticity and presence upon subjects according to hyper-scanning techniques using in neuroscience (Montague et al., 2002; Ma et al., 2011).

According to our ERP measures, larger amplitudes of parietal P300 were elicited of winning (SW and CW) compared with losing (SL and CL) situation (see Figure 5). As mentioned by Yeung and Sanfey (2004), P300 was sensitive to the reward value of alternative, non-selected stimuli (Yeung and Sanfey, 2004). P300 was also thought to reflect the magnitude of rewards or favorable outcomes (Yeung and Sanfey, 2004; Sato et al., 2005; Luo and Qu, 2013). A more favorable split generated a larger P300 than an unfavorable one (Wu et al., 2011, 2012). In addition, larger P300 amplitudes revealed positive outcomes than negative ones (Hajcak et al., 2004; Yeung et al., 2005; Peterburs et al., 2013). There were no monetary gains or losses in our research design, however, the simple results of win and lose activated subjects' reward area of brain and brought emotion changes as well. It might reveal that subjects exactly “loved to win and hated to lose” through ERPs evidences. Furthermore, larger amplitudes of late positive potentials (LPP) were evoked in CW and CL results compared to SW and SL situations (see Figure 5). The latter portion of ERP waveform LPP indicated elevated positivity to high-arousing stimulation according to Cuthbert et al. (2000). Cuthbert et al. (2000) in our study, when subjects had continuous win or continuous lose experiences, higher arousing emotion intensity had been elicited compared with single win or single lose experiences. Gajewski et al. (2016) used facial expression as stimuli in their study to analyze the neural correlates of a simulated purchase decision task. However, no significant effects were found by facial expression upon participants' neural process. The explanation might be those facial pictures were irrelevant with the shopping task in their experiment. In our study, we simulated a virtual online interactive game (FP) developed by the shopping website and gave a good reason (would receive coupons for next shopping) to subjects to accept playing the game before browsing products. In this context, FP priming well combined with the adding to cart task. As a result, the emotional reflection of FP results made a significant influence upon subjects' perception of products price and their behavior.

According to the preference rating scores of the selected hard-discs, it was revealed that subjects' attitudes regarding the products without price information were discrete. They made a decision whether adding the hard-disc to cart or not only depending on the difference price information or their intuition. Knutson et al. (2007) suggested that consumers mostly avoided excessive prices products in addition to those deeply preferred ones (Knutson et al., 2007). In our case, subjects were assumed to choose cheaper hard-discs as all the products properties were nearly the same. Interestingly, subjects' choices were impacted to a great extent by different arousal emotions when continuous winning or continuous losing in the FP game induced. The adding to cart proportion of CWHP (*M* = 55.1%, *SD* = 0.36) was higher than CLHP (*M* = 20.29%, *SD* = 0.24). Meanwhile, the adding to cart proportion of CWLP (*M* = 77.1%, *SD* = 0.29) was much higher either compared with CLLP (*M* = 48.6%, *SD* = 0.31). It was easily to understand that higher adding ratio of low price hard-discs than high price ones. When we compared the adding proportion in same emotion situations (CW or CL), low price hard-discs were added to cart more than high price ones. However, the adding ratios of either high price or low price hard-discs were higher in CW situation than in CL situation (see Figure 4). It might be clearer to explain this phenomenon through the event-related potential measures. According to the visual observation and statistical analysis, two main ERP components—P2 and LPP were found when we compared CWHP and CLHP conditions. Standard ERP recording methods indicated that P2 component was sensitive to the onset of pleasant-going arousal-related amplitude modulation persisting until stimulus offset (Carretié et al., 2001a,b; Amrhein et al., 2004; Olofsson and Polich, 2007; Olofsson et al., 2008). Kamei et al. (2018) also observed that the auditory P2 and the occurrence of pleasant emotions were higher in the winning streak (WS) condition than in the losing streak (LS) condition (Kamei et al., 2018). Similar to Kamei et al. (2018), a larger P2 peak-amplitude from 150 to 220 ms was observed in CWHP compared with CLHP condition, might because CW elicited more positive emotions than CL and this emotion state was lasting then continuously influenced subjects' price perception resulting higher adding ratio of CWHP than CLHP (see Figures 4, 6). In addition, larger LPP amplitudes had been observed in CWHP compared to CLHP either (see Figure 7). Hajcak et al. (2010) concluded that emotional influences on the LPP related to the emotional intensity of stimuli (Hajcak et al., 2010). In our study, high price hard-discs corresponded with CW condition elicited larger LPP amplitudes than CL condition. CW aroused a more intense emotion than CL and this highly arousal emotion lasted when products with price onset then changed subjects' price perception and finally caused a higher adding ratio of CWHP. The adding to cart proportion of CWLP was higher than CLLP as well on the basis of the behavioral data. In ERP measures, we found a significant difference in latencies of peak amplitudes during the time window 500–800 ms between CWLP and CLLP. The peak amplitudes of late positive potentials occurred earlier in CWLP (*Mean*_*CWLP*−*LPPLatency*_ = 609.45 ms, *SD* = 13.482) than CLLP (*Mean*_*CLLP*−*LPPLatency*_ = 662.37 ms, *SD* = 12.636) condition (see Figure 7). That might explain why higher adding ration of CWLP compared with CLLP.

However, we did not find effective ERP components when we examined other conditions (CWHP vs. CWLP, CLHP vs. CLHP). This further illustrated that in the same emotion condition, subjects made decisions based on their common sense and had more dependence on their past experiences.

The findings of this study indicated that emotions indeed played an important role in consumers' price perception and influenced their buying behavior. Additionally, the new experimental paradigm we creatively used could be applied in other marketing research issues. Win or lose feelings might impact not only customers' price perception, but also their other purchasing processes. However, the observation of brain-behavior relationship of our study was under well-controlled laboratory conditions and the ecological validity of the research might be limited. The customers' actual buying behavior might be dissimilar from our experimental settings. In the real marketing environment, the decision process of purchase might be more complicated and the information searching time might be much longer. There were some areas needed to improve in our future research. Firstly, the design of experiments needed to better reflect participants' real buying processes. Secondly, studies on how continuous win effect influenced consumer's buying behavior needed to be applied in other marketing issues, such as brand preference, product design, advertising etc. Moreover, mobile EEG equipment could be adopted to record customers' brain activities outside the laboratory but in more natural situations (Bleichner et al., 2015).

## Conclusion

To sum up, this study creatively integrated a competitive game without monetary gains or losses as an emotional priming paradigm into the investigation of how emotions influencing subjects' price perception. Both behavioral and ERP results indicated that subjects' price perception was deeply impacted by emotions. Emotions induced by winning include Single Win and Continuous Win were more positive than those evoked by losing include Single Lose and Continuous Lose situations. Continuous Win and Continuous Lose aroused more intensity emotions compared to Single Win and Single Lose. These emotion states lasted when hard-discs with price presented to subjects and evoked P2 and LPP components in CWHP and CLHP conditions as well as latency differences between CWLP and CLLP conditions. Thus, subjects added more hard-discs to cart when they experienced continuous win results compared to continuous lose situation. It was verified that emotion played an important role in consumer behavior and marketing from a new angle. Moreover, we firstly proposed this phenomenon as a new concept—“Continuous Win Effect” and planned to do more relative researches on this topic. Nevertheless, there still were some limitations in our research. First of all, the experimental setting might be very different from consumers' actual buying behavior. In the real marketing place, the decision process of purchase might be more complicated and the information searching time might be much longer. Secondly, we only tested the results of winning and losing without considering the situation of draw and others. Third, we only examined price perception of a well-known electronic product—mobile hard-disc without considering other products such as luxury goods, furniture, foods and beverages etc. The question might be whether emotions could impact price perceptions and consumers' behavior on these products as well or not. These deficiencies would be improved in future researches.

## Ethics statement

As corresponding author, Qingguo MA confirmed that all experiments were performed in accordance with relevant guidelines and regulations. All experiments were approved by the Neuromanagement Laboratory Ethics Committee at Zhejiang University and reviewed by the ethics committee. The research design respects the personality of the subjects and will not cause psychological and physical damage to the subjects. The experiments are performed in accordance with APA Ethics Code and the principle of <International Ethical Guidelines for Biomedical Research Involving Human Subjects> by CIOMS. Further, written informed consent was obtained from all participants prior to the ERP experiments.

## Author contributions

QM designed the experiments. LZ and MW prepared the experiments and collected behavioral and ERP data. LZ processed and analyzed data. QM and LZ wrote the main manuscript text and prepared all the figures. All authors reviewed the manuscript.

### Conflict of interest statement

The authors declare that the research was conducted in the absence of any commercial or financial relationships that could be construed as a potential conflict of interest.
