# Corrigendum: “You Win, You Buy”—How Continuous Win Effect Influence Consumers' Price Perception: An ERP Study

**DOI:** 10.3389/fnins.2019.00167

**Published:** 2019-03-05

**Authors:** Qingguo Ma, Linanzi Zhang, Manlin Wang

**Affiliations:** ^1^School of Management, Zhejiang University, Hangzhou, China; ^2^Institute of Neuromanagement Science, Zhejiang University of Technology, Hangzhou, China; ^3^Business School, Ningbo University, Ningbo, China; ^4^Academy of Neuroeconomics and Neuromanagement, Ningbo University, Ningbo, China; ^5^School of Management, Guizhou University, Guiyang, China

**Keywords:** continuous win effect, price perception, event-related potentials, P2, P300, LPP, neuromanagement, neuromarketing

There is an error in the Funding statement. The correct number for the “National Project” is “AWS14J011”.

The authors apologize for this error and state that this does not change the scientific conclusions of the article in any way. The original article has been updated.

